# Representative Random Sampling of Chemical Space

**DOI:** 10.1021/acs.jctc.5c01523

**Published:** 2025-12-02

**Authors:** Diego J. Monterrubio-Chanca, Guido Falk von Rudorff

**Affiliations:** † Institut für Chemie, Universität Kassel, 34109 Kassel, Germany; ‡ 9178 Center for Interdisciplinary Nanostructure Science and Technology (CINSaT), Heinrich-Plett-Straße 40, 34132 Kassel, Germany

## Abstract

An overwhelming majority of molecules remain unexplored. This is mostly due to the sheer number of them, which prohibits any enumeration of chemical space, the set of all such molecules. In practice, only subsets of chemical space are considered, but those subsets exhibit substantial bias, prohibiting the data-driven characterization of chemical space itself. In this work, we provide a method to produce unbiased representative random samples of the chemical space without enumeration of constituent molecules and to estimate the number of molecules in any custom chemical space. The approach is applicable to molecules that can be represented as graphs and runs efficiently even for molecules of 30 atoms. We use it to estimate the representativeness of current databases with respect to their underlying chemical space and establish a necessary criterion for a lower bound of database sizes to be representative of an underlying chemical space.

## Graphical abstract

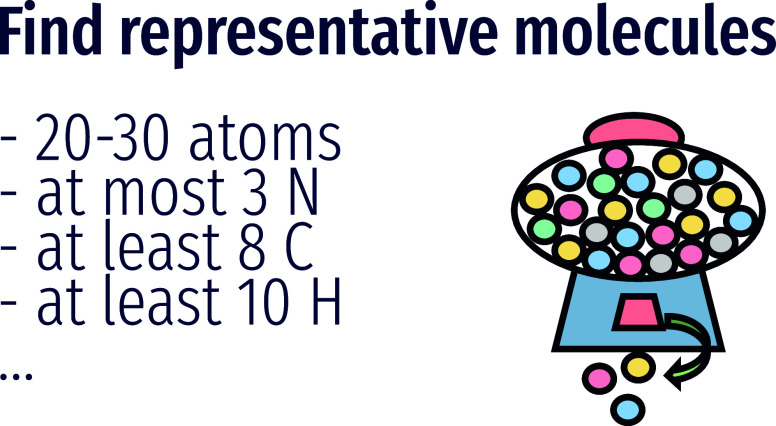

1

Chemical space is the set of all stable compounds from which we can choose molecules of favorable properties and therefore the search space for compound design.^1–4^ For practical reasons, we typically consider only a subset of that space, even when statements about general structure–property relationships are made. If this subset is not representative of chemical space, it introduces a bias that propagates to the conclusions drawn from the subset analysis. Although quantum chemistry data sets^5–19^ grow larger and larger,^20,21^ any self-similarity of the data points they cover does not necessarily reduce the bias of the data distribution.^22,23^ These self-similarities are also introduced by the whole tool chain that is used in generating the databases.^24,25^ Therefore, it is highly desirable to assess the chemical space in a provably unbiased manner. Unbiased exploration would improve our intuition on chemical processes and modeling. Textbook concepts such as electron-donating/withdrawing groups, the Hammond postulate,^26^ or Hückel’s rule^27^ are derived from experimental data (which by the nature of the intricate experimental process is self-similar) or theoretical models. As such, this might constitute overfitting toward the tiny section of chemical space we have observed so far and may break down systematically for other regions,^28–31^ failing to generalize to an appreciable number of systems.^32^

Considering the main goals of modern machine learning methods, that is, efficiency, accuracy, scalability, and transferability (EAST),^33^ unbiased reference data connects efficiency and transferability. The smaller the required amount of training data, i.e., the higher the data efficiency, the more evenly balanced this data is over the target domain, and the higher the transferability which can be reasonably expected.

Exploring the vastness of chemical space is relevant for a wide range of applications, such as drug design^34,35^ or catalysts,^36–38^ novel materials,^39,40^ and molecular property prediction.^41,42^ Given the extent of exponentially increasing chemical space, its vast majority is unexplored and probably will remain unexplored, which makes it even more important to traverse the options most efficiently. Researchers have addressed this substantial challenge by forward design based on predicting individual molecular properties with a wide range of methods^43–47^ and inverse design using generative models.^48,49^ In forward predictions, a core challenge is to reliably assess the generalization error, which is typically done using hold-out data sets, assuming that the underlying database is representative of the problem space since, otherwise, aggregate measures do not apply to true out-of-sample data. For generative models, an open question is whether their image space captures the full target domain, whether they are most likely to reproduce compounds similar to the training data, or how to restrict to meaningful output spaces.^50,51^ While large training data sets were a necessary requirement for machine learning of molecular properties, most early data sets make assumptions simplifying their generation and include artificial biases toward compounds which have better support from cheminformatics toolchains^11^ or quantum chemistry software.^52^ Those restrictions, however, skip interesting systems which would commonly be assumed to be unstable, such as cubane,^53^ hexamethylbenzene dication,^54^ argon fluorohydride,^55^ hexanitrogen,^56^ and C_48_ (cyclo^48^ carbon).^57^ This impacts or hinders the discovery of the so-called unknown unknowns, i.e., new effects or physical phenomena.

One approach to systematic exploration of chemical space is based on full enumeration. Over decades, many tools have been developed, such as MOLGEN,^58^ OMG,^59^ PMG,^60^ surge,^61^ Maygen,^62^ and enu.^63^ What they have in common is that they systematically enumerate the space of all feasible molecules by constraining themselves to those bond topologies that are in line with standard valence bond rules and then systematically generate molecular graphs. The major achievement of those codes is that duplicate checks are not required, as the generation itself already guarantees uniqueness. However, even those approaches are limited in molecular size since chemical space is scaling even further than the few tens of atoms that can commonly be treated. To combat the exponential scaling of chemical space, most of these tools allow the definition of further restrictions, such as avoiding certain features of molecular graphs or further constraining bond topology or subgroups. This has been very helpful in practical application but only marginally increases the size of the molecules that can be enumerated systematically while introducing the aforementioned biases. In the context of structure elucidation, stochastic approaches have been pursued^64^ with a focus on identifying possible target compounds if molecular fragments are known, which does not allow for random sampling of a chemical space. More recently, larger chemical diversity became the goal of molecular generators.^65^

Moreover, these methods cannot be parallelized easily, even though some have been developed with parallelization in mind.^60,61^ Since in the systematic enumeration the problem cannot be subdivided evenly, even approximately random uniform sampling is infeasible with those methods, unless generating a complete list of all molecules would be feasible.

The infeasibility of full enumeration renders it necessary to employ probabilistic approaches for unbiased predictions over a chemical space constrained by domain-specific physical limits such as element variety, molecular size, and stoichiometries instead of software support or practical convenience. For example, local refinement in chemical space can be carried out reliably using Monte Carlo methods.^66^ However, global exploration poses a considerable challenge: accessing all valid molecules requires long, sequential transitions, which are computationally expensive and difficult to parallelize. Ensuring detailed balance and unbiased sampling further complicates the process, leaving full exploration through classical Markov Chain Monte Carlo (MCMC) impractical. An unbiased random sampler, however, could parallelize the MCMC by providing a more diverse set of starting points.

In this work, we present a method that generates approximately uniform random samples spanning a chemical space. We only require that the chemical space admits a representation of a molecule as a finite molecular graph, which excludes essentially the same application domains where vanilla SMILES representations are inapplicable, such as polymers.^67^ This approach enables scalable and representative exploration of the chemical space, suitable for applications in design, learning, and structural analysis.

## 2 Methods

Our method, representative random sampling (RRS), produces a sequence of molecules that are approximately uniformly randomly selected from a given chemical space spanned by all molecular graphs that satisfy valence bond rules. While this is not applicable to all chemical spaces, it is commonly used.^67^

In RRS, we consider atoms of different valences as different atom types; e.g., phosphorus of valence 3 and phosphorus of valence 5 would be different atom types. The atom types form the set *A* of pairs (*e*, *v*) with element *e* and valence *v*. All atoms of the same valence are of the same valence type, e.g., hydrogen and all halogens. In the following, *V* ≡ {*a*_2_|*a* ∈ *A*} is the ordered set of valence types. For each valence type *v*, there are potentially multiple atom types *A*(*v*) ≡ {*a* ∈ *A*|*a*_2_ = *v*}, counted by valence-type multiplicity |*A*(*v*)|. This allows us to simplify the problem considerably, since the number of possible molecules that can be formed given a set of atoms only depends on their valence type, not on their element. While enumerating graphs via graph counting methods is implemented in many codes, it is not fast enough for the problem size at hand, where computing or storing the list of molecular graphs is completely infeasible. We address this by first estimating the total number of molecular graphs for each sum formula within a search space and then uniformly randomly sampling from that search space by selecting a chemical formula, followed by a Markov Chain Monte Carlo sampler within that chemical formula. The key difficulty here is to reliably estimate how many molecular graphs exist for any given chemical formula.

### 2.1 Obtaining All Chemical Formulas

In principle, obtaining all chemical formulas within a chemical space is an integer partition problem where a certain number of atoms is distributed over all possible elements allowed in the chemical space. Due to the sparsity structure of those partitions, a nested approach is substantially more efficient than direct partitioning. We first find the set of constitutions *C* for molecules in a given chemical space by repeated integer partitions. A single constitution is defined as a multiset of atom types, i.e., elements and valencies.

Let *p*(*c*, *B*) be the integer partition of the ordered set *B* into *c* parts {(*x*_1_, ..., *x*_|*B*|_)|∑_*i*_ *x*_*i*_ = *c*}. For each possible number of atoms *N*_a_ in the chemical space, we obtain a set of partitions

1 $$O\left(N_{a}\right)\equiv \left\{q\in p\left(N_{a},V\right)|M_{V}\left(q\right)even\wedge S\left(q\right)\right\}$$

where *M*_V_(*q*) ≡ ∑_*i*_ *q*_*i*_ *v*_*i*_ is the sum of all degrees and *S*(*q*) is true of all bonds that can be saturated given the valencies.^61^ *M*_V_(*q*) needs to be even such that a graph is feasible. At this stage, only the valencies are taken into account but not the valence type (e.g., so far monovalent hydrogen and fluorine would not be distinguished).

We now find all possible constitutions *C*(*N*_a_) by taking the Cartesian product *c* of all partitions within each valence type, i.e., by finding all constitutions including the atom type information

2 $$C\left(N_{a}\right)\equiv \underset{i}{\cup }\left\{c\left(x\right)|x\in O\left(N_{a}\right)\right\}$$

3 $$c\left(x\right)\equiv \prod_{i}\left\{a^{y_{i}}|y_{i}\in p\left(x_{i},A\left(v_{i}\right)\right)\wedge a\in A\left(v_{i}\right)\right\}$$

This two-stage approach is computationally much more efficient yet otherwise identical to directly partitioning *N*_a_ atoms into the corresponding elements from the chemical space, since only unique cases are generated: e.g., CF_4_ and CH_4_ correspond to the same element in *O*(5) but become different constitutions in *C*(5).

For each constitution, there are potentially many connected loop-free multigraphs in which the vertices are labeled with the chemical element. These graphs all have the same degree sequence *d*, where each vertex (an atom) is also assigned an element. To exploit symmetries (such as the CF_4_ case above), we only consider unique protomolecules where fictitious element labels are placeholders for all elements of compatible atom types, but including the distinction from the constitution (see Figure 1). For example, using superscripts as valencies, CH_2_F_2_ and CH_2_Cl_2_ both form protomolecules of family X^4^Y_2_^1^Z_2_^1^ and, therefore, are distinct from CF_4_ and CH_4_, which belong to protomolecule family X^4^Y_4_^1^.

**Figure 1:**
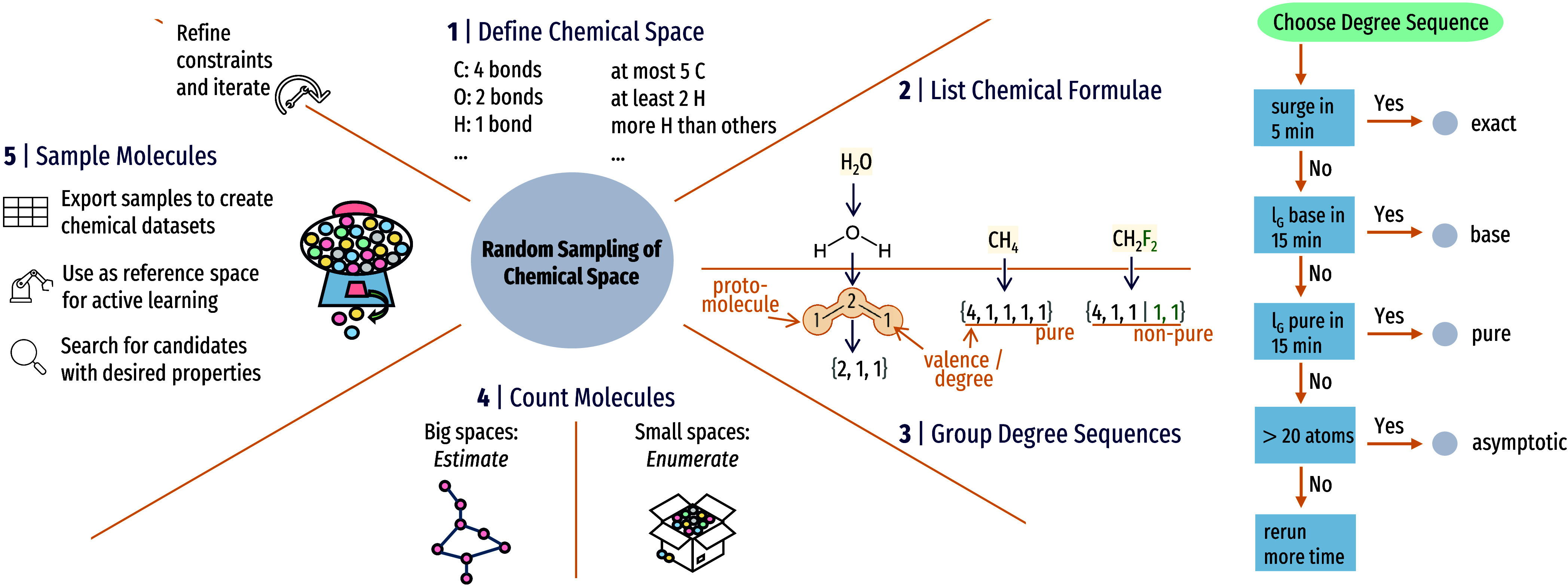
Overview of the random sampling procedure and applications. Left: From a well-defined chemical space based on valency and stoichiometry restrictions (1), all chemical formulas are obtained by integer partition (2) and grouped by degree sequence (3). Here, we distinguish between a concrete molecular graph without element information (protomolecule) and the sequence of atom valences, i.e., the degree sequence. The latter may or may not contain several elements of identical valency, forming nonpure and pure degree sequences, respectively. For each chemical formula, the total size is estimated (4), which allows for appropriately weighted random sampling (5). Right: Details of step 4 (counting molecules), which is part of building the database, not part of sampling random molecules (step 5). For a given degree sequence, we first try exact counting (surge) and resort to heuristics based on the average path length *l*_G_ of decreasing accuracy once a time budget is exhausted.

### 2.2 Estimating Molecule Counts

For a small number of atoms (typically up to about 10), direct systematic enumeration of all protomolecules is feasible. In this work, we use a surge;^61^ all resulting counts are distributed with our software packages nablachem. Beyond this realm, we obtain approximate counts by using results from network topology. First, we introduce a new graph, the universe *U*(*d*), which has one vertex for each protomolecule that can be obtained from a labeled degree sequence *d*. In this universe, two vertices are connected if and only if their corresponding vertices have a minimal graph edit distance. Typical molecular graphs can be modified into many different other molecular graphs by equally simple edits (e.g., the switch of two edges connecting for different atoms, a minimal change), which would make this universe a network of high clustering coefficients. Networks of high clustering coefficients are also called small-world networks, which are known^68^ to exhibit a relation between the average length *l*_G_ of the shortest path between two vertices and the total number of vertices in that graph

4 $$l_{G}∼\log\left|U\left(d\right)\right|$$

This assumes that molecular universes can be considered a small-world network, which numerical results will justify. The main advantage of this approach is that we only need the average distance between elements of this universe, meaning we do not need to enumerate all of the molecules that have the same labeled degree sequence but rather a tiny subset thereof since the average of their pairwise distances converges quickly. This is the key step enabling reliable estimation of molecular counts in this work. Obtaining *l*_G_ by randomly sampling protomolecules and finding their edit distance then allows us to estimate the total number of protomolecules |*U*(*d*)| for that labeled degree sequence *d*. This is computationally feasible until about 30 atoms.

For any pure degree sequence, i.e., where the maximum valence multiplicity is 1 (see Figure 1), the number of distinct protomolecules is minimal among all degree sequences which have the same valencies but with larger multiplicities, e.g., if we have two atoms of the same valency and identical element label (pure degree sequence) that yields one protomolecule. If we have two atoms of the same valency and different element labels (nonpure degree sequence), we can now have three different protomolecules (with the element labels one–one, one–other, and other–other). If we assume that for large and complex molecules which contain many branches and asymmetries, isomorphisms are rare, we expect that almost always each atom site is unique in the chemical environment. Therefore, the number of possible protomolecules *N*_P_(*d*) upon introducing more elements with the same valency can be approximated by applying combinatorial counting to groups in the labeled degree sequence *d*

5 NP(d)=∏v∏i(∑j≥icjci)

where *v* runs over all valencies in the degree sequence, and *i* and *j* run over all elements of that given valency *v*. For each *i*-th element, *c*_*i*_ is the number of atoms of that element. Note that for unlabeled degree sequences, *N*_P_(*d*) = 1. The asymptotic behavior of protomolecule counts^69^ suggests that the total number of protomolecules should be inversely related to the total number of bonds *M*(*d*). This yields

6 $$l_{G}\left(d\right)=\left(1+{\left[\sum_{i}d_{i}\right]}^{-1}\;\log\;N_{P}\left(d\right)\right)l_{G}\left(d_{U}\right)$$

as an expression to estimate the average path length *l*_G_(*d*) for an arbitrary degree sequence if the average path length *l*_G_(*d*_U_) for the corresponding pure degree sequence is known. The number of protomolecules is then obtained from *l*_G_ as it is for smaller molecules. This is computationally feasible until about 30 atoms.

Finally, we have reached the realm of asymptotic scaling, where formal statements about the number of protomolecules exist.^69^ This allows the scaling behavior of a quantity proportional to *l*_G_ of any unlabeled degree sequence. Using the permutation logic of the previous paragraph allows us to then obtain *l*_G_ for any labeled degree sequence. This is extremely cheap and can be applied to arbitrarily large molecules. The bottleneck then becomes the enumeration of all sum formulas from the integer partitions in the first place.

### 2.3 Estimating the Minimal Graph Edit Distance

The average path length of the shortest path between two vertices in the universe is the graph edit distance, meaning the minimal sets of edits that have to be done to one molecular graph in order to obtain the other. While the graph edit distance cannot be determined in polynomial time, efficient heuristics exist. In this work, we measure the graph edit distance as the minimal Wasserstein metric between all of the permutations of the adjacency matrices of two molecules. For small molecules with fewer than 100 permutations, all permutations are considered, so the minimization is exact. For larger ones, we use the minimal Wasserstein metric obtained from six different heuristics: (i) random shuffling, (ii) repeatedly search for a pairwise switch of atom indices which minimizes the metric from a random initial value, (iii) treating the permutation as a quadratic assignment problem (QAP) with the 2-opt^70^ algorithm allowing all permutations, (iv) as a repeated QAP with permutations freezing all but one chemical element sequentially in one scan, (v) as a QAP with permutations freezing all but one chemical element in multiple scans, and (vi) employing a depth-first (DFS) graph edit distance optimizer.^71^ Each such heuristic has been called about 50–100 times, depending on the graph size. For the cases considered in this work, all heuristics typically finish within 15 min on a single core. The work can be parallelized trivially since all degree sequences are fully independent. We empirically find that for different parts of chemical space, these heuristics have different efficiencies, but we did not analyze this further as the current computational cost is acceptable, especially given that this heuristic needs to be done only once and can be reused even if different element labels are required. We generally expect random shuffles to be efficient for almost symmetric graphs, sequential switches to be efficient for tree-like topologies, QAPs to be efficient if many different elements of the same valency are included, and DFS to be the generally suitable baseline. Therefore, it may be computationally more efficient to balance the computational budget between those methods depending on, e.g., the number of cycles admitted by a given degree sequence, should resource constraints require this. Extending the current database is necessary only if more elements of the same valency are supposed to be found in the same small molecule: otherwise, the pure approximation and ultimately the asymptotic scaling would suffice.

## 3 Results

### 3.1 Estimating Molecule Counts

The core idea to estimate the size of chemical spaces from the average path length following the small-world network results in eq 4 first needs validation. To this end, we enumerated small molecular graphs using surge.^61^ Table 1 shows the chemical spaces considered, and Table 2 shows the sizes of the molecules for which there is precomputed data available. It is important to note that the element labels are arbitrary in this work: one can easily replace one element forming a single bond with another element, forming a single bond without any additional computation. The precomputed databases are limited by how many distinct elements can be used of a given valence, the valence multiplicity.

**Table 1:** Chemical Spaces Used in This Work^a^

space	valence	multiplicity	example
A	1	5	F, H, Cl, Br, I
	2	2	O, S
	3	2	N, P
	4	3	C, S, Si
	5	2	N, P
	6	1	S
B	1	5	F, H, Cl, Br, I
	2	2	O
	3	2	N, P
	4	1	C
	5	1	P

a Element labels are illustrative only since any labeling of equivalently bonding elements yields the same protomolecule set.

**Table 2:** System Size Coverage of the Data in This Work for Each Chemical Space^a^

space	exact	base	pure
A	3–10 atoms	10–15 atoms	10–20 atoms
B	3–10 atoms	10–22 atoms	10–31 atoms

a For the domain labeled exact, the correct weights are known from enumeration, so the sampling is guaranteed to be uniform. For base data, the average path length has been found heuristically from explicit sampling of all labeled degree sequences. For pure domains, this has been done for all unlabeled degree sequences only. Above the specified domains, asymptotic scaling relations are employed. Base is a strict superset of pure data.

The nablachem.space^72^ Python package contains all this precomputed data and automatically chooses the most accurate method available when queried. It also contains the code to extend the databases in this work to arbitrary chemical spaces.

Figure 2A shows the correlation between the number of molecules *n* for a given chemical formula and the average path length *l*_G_ as determined by our heuristic. We find that there is a strong correlation between log­(*n*) and *l*_G_, indicating that eq 4 is applicable to the data at hand. This is a key result which allows us to estimate the number of molecules for a given chemical formula without any enumeration by parameterizing the proportionality from eq 4. When fitting to the exact counts for all 285,656 unique degree sequences covering 26,076,359,902,577 molecular graphs in our database in Figure 2A, we obtain the following approximation with *R* ^2^ = 0.61

7 $$\log\left|U\left(d\right)\right|\approx 1.220l_{G}-0.7295$$

**Figure 2:**
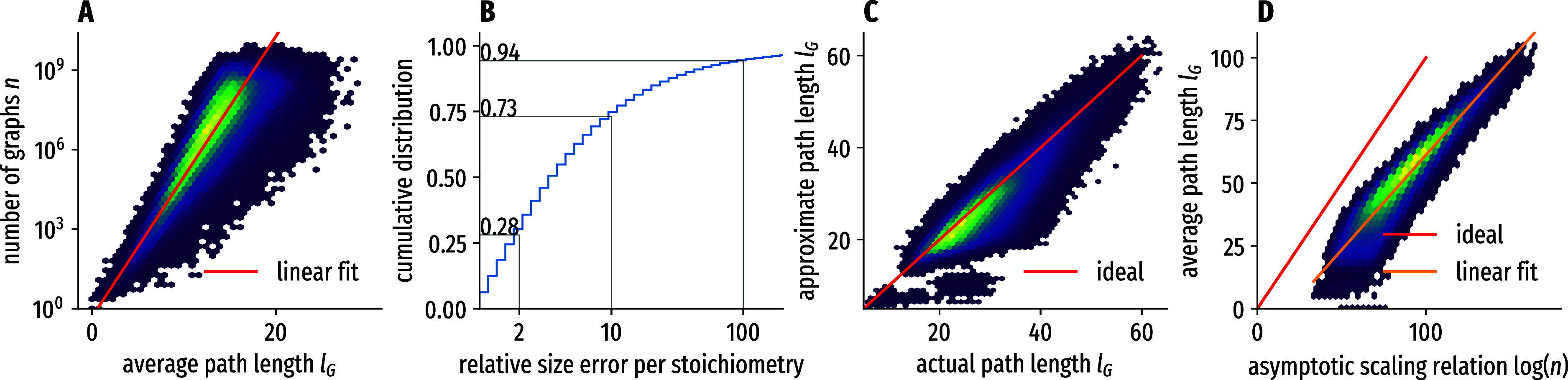
Accuracy of the correlations exploited in this work. (A) Obtaining counts *n* of protomolecules from the average path length *l*_G_ in the universe graph for all 285,656 stoichiometries with exactly 26,076,359,902,577 molecular graphs in our database. Histogram of the correlation between *l*_G_ and *n* together with the expected linear fit. (B) Relative error of the estimated and the actual number of graphs for a given stoichiometry as a cumulative histogram: correct order of magnitude is reached for 73% of the cases. (C) Estimated average path length vs actual average path length for all 553,132 nonpure labeled degree sequences in our database. Note that there is no fitting involved in this step. (D) Accuracy of estimating the logarithm of the number of molecules with pure degree sequences from calibrating the asymptotic scaling relations in eq 8. The density histogram covers all 148,620 pure degree sequences with more than 20 atoms in our database.

We find that extending the sampling steps of our heuristic does not reduce the average path length significantly. We therefore consider the deviations in the histogram to be the consequence of the extent to which the small-world network approximation is valid, especially for the residuals, where the average path length seems to be below what would be expected from the fit. For the opposite side (a larger average path length than suggested by the fit), we observe larger residuals, indicating that the heuristics may be inefficient in individual cases. Besides larger stoichiometries being harder to quantify, we do not observe any trends. Since the fit is performed on logarithmized data, it is well-balanced across the widely different number of molecular graphs in each stoichiometry: Figure 2B shows the histogram of the relative errors made in the size estimation between the real enumerated number of graphs and the estimate from eq 7. Our method estimates the correct order of magnitude for each stoichiometry in 73% of the cases. For small stoichiometries for which exact molecule counts are known, there is no error in size estimation and, therefore, no bias in the resulting sampling, since the sampling of individual graphs for a given stoichiometry is uniform (see Figure 3). For stoichiometries with estimated sizes, an individual bias remains: there is a systematic error for any estimated single stoichiometry, which does not average out. Since the residuals in Figure 2A are not generally biased toward over- or underestimation of the molecular graph count, aggregate counts of large chemical spaces are expected to be similarly truthful as the distribution for the individual counts in Figure 2B, as per the law of large numbers.

**Figure 3:**
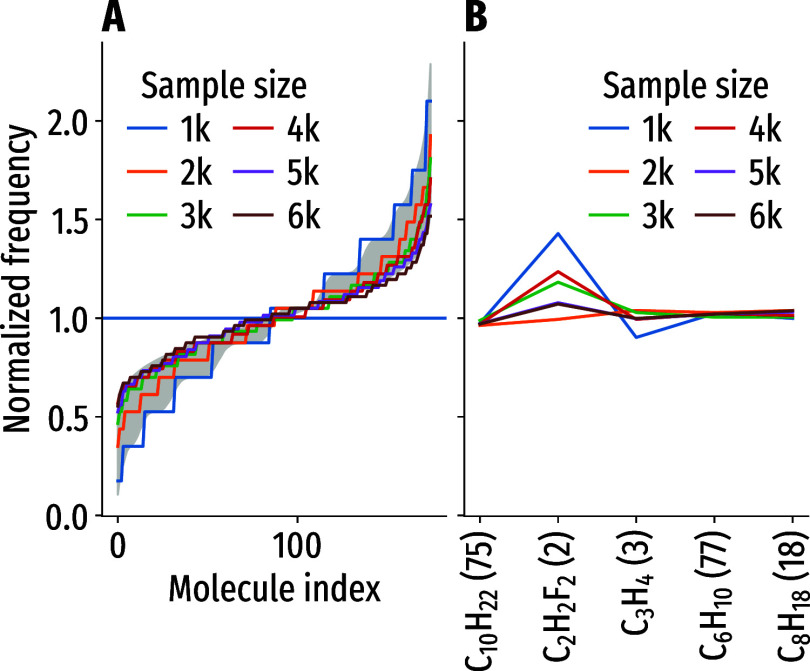
Tests for uniformity of the sampling results for joint sampling from several stoichiometries with 175 molecules in total. All data-normalized s.t. unit frequency is uniform sampling. (A) Sorted sampling frequencies for individual molecules for different sample sizes. Expected limits for perfectly uniform sampling are shown as a gray area; one limit is expected for 1000 samples, the other for six times as many. (B) Sampling frequency for stoichiometries normalized by the number of molecular graphs for each. The expectation for perfect uniform sampling is constant unit frequency.

Since at some point even enumerating all degree sequences of interest becomes prohibitively expensive, eq 5 was the consequence of a simple combinatorial argument for estimating the number of unique graphs arising from nonpure degree sequences if only the size of the pure degree sequence (or, equivalently, its average path length) is known. This hypothesis is probable, since a random molecular graph is unlikely to be symmetric, and could be confirmed in the data: Figure 2C shows the correlation between the average path length using the explicit minimum edit graph heuristic above and the results of applying eq 5. Note that no fitting was involved in this step. This greatly reduces the computational complexity from estimating the size of all degree sequences to estimating the size of pure degree sequences, of which there are many fewer.

Going toward even larger molecules, the heuristic determination of the average path length becomes prohibitively expensive. Fortunately, asymptotic scaling relations for the number of graphs in this case are available^69^ (Theorem 1.1) and are reproduced here for simplicity

8 $$\begin{array}{ll} G & =\frac{M!}{\left(M/2\right)!2^{M/2}k_{1}!\cdot \cdot \cdot k_{n}!}\\ & \exp(\left(y_{1}-\frac{1}{2}\right)\frac{M_{2}}{M}+\left(x_{2}-\frac{1}{2}\right)\frac{M_{2}^{2}}{2M^{2}}+\frac{M_{2}^{4}}{4M^{5}}\\ & -\frac{M_{2}^{2}M_{3}}{2M^{4}}+\left(x_{3}-x_{2}+\frac{1}{3}\right)\frac{M_{3}^{2}}{2M^{3}}+O\left(k_{\max}^{3}/M\right)) \end{array}$$

9 $$M_{r}=\sum_{i}^{n}{\left[k_{i}\right]}_{r}$$

where *n* is the length of the degree sequence **k**, [·]_r_ denotes the falling factorial, and *x*_*i*_ = 1 if *i* is allowed as nonloop edge multiplicity (otherwise 0) and *y*_*i*_ = 1 if *i* is allowed as loop-edge multiplicity (otherwise 0). Here, this means all *x*_*i*_ = 1 and all *y*_*i*_ = 0 for *i* > 0.

Since this is an asymptotic scaling relation, the prefactor is unknown. In this work, we calibrate the scaling relation to actual data by a linear fit of *l*_G_ to log­(*G*) on all 148,620 pure degree sequences with more than 20 atoms for which *l*_G_ is known (see Figure 2) and obtain

10 $$l_{G}\approx 0.7561\;\log\left(G\right)-14.40$$

this simple model performs remarkably well and remains feasible as long as listing the integer partitions of the total number of atoms is affordable. The nonpure degree sequence count is then estimated using eq 5 as before.

When adding *t* hydrogens (or other monovalent elements) to a fixed stoichiometry, eventually the number of molecular graphs has to decrease as the double bond equivalents of the graphs approach zero. The asymptotic scaling relation does not take this effect into account: it would grow monotonously instead, even once graphs become infeasible due to the negative double bond equivalent. Comparing to the cases for which we know the exact graph count from our database, we find that the relation tends to overestimate by *t*!, so we correct it by this amount. Naturally, the extreme end of the molecule count estimates is impossible to calibrate, so we recommend limiting the use of the asymptotic scaling relation once it yields a large variance for comparable stoichiometries.

### 3.2 Sampling Molecular Graphs

Once for each chemical formula the total number of molecular graphs is estimated, we can finally sample molecules for a particular chemical formula, which has been selected with a probability proportional to its size in the selected chemical space. We implement this with a Markov–Chain–Monte Carlo (MCMC) approach, which allows us to obtain a random connected loop-free undirected multigraph of a given degree sequence once a single such graph for the given degree sequence has been obtained, which we obtain from a (biased) stub-based random graph generator.^73^ This is preferable over using MCMC generation over all (including disconnected) graphs which is available for fixed degree sequences,^74,75^ since this also works for the limit of trees (e.g., branched alkanes), which has degree sequences for which almost all random graphs are disconnected. Since the space of molecules from which to sample is restricted to those of the same degree sequence, sampling the distribution converges quickly even for large molecular graphs.

First, we verified the uniform sampling behavior. Figure 3 shows the results of a test case, where five stoichiometries with 175 molecular graphs in total are sampled jointly at random. For this case, we can verify the uniform sampling since we know all possible outcomes and can easily obtain many more samples than possible molecules. First, we consider the sorted frequencies of molecular graphs drawn at random from all five stoichiometries together. For uniformly drawn discrete samples, this creates a characteristic s-shaped curve which becomes flatter with increasing sample size. Figure 3A shows this expected curve in gray for the smallest sample size and the largest sample size (1000 and 6000, respectively). The actual distribution of the sorted counts follows the theoretical expectations, both for the small and large sample sizes. In Figure 3B, we confirm that the frequency of the five stoichiometries follows the number of their respective molecule counts: the data is normalized such that a frequency of 1 denotes perfectly uniform sampling. This confirms that disconnected regions in chemical space are indeed sampled uniformly at random.

The sampling code is available in nablachem.space,^72^ and it can also be run interactively on random-molecule.org.

Given that large-scale databases of quantum chemistry data are systematically generated in a high-throughput setting, it is just natural that some artifacts of the tooling involved affect the exhaustiveness of the result. For example, the widely used QM9 database,^11^ which is built to almost exhaustively contain molecules from C, H, N, O, and F with ≤9 heavy atoms, does not contain benzene, carbon dioxide, ethylene, butadiene, and other common compounds, even though they would belong to the target space of the database.

With our method, we can estimate whether the given database is likely to be representative of the underlying chemical space by comparing the distribution of molecules from the underlying chemical space with the distribution of the database entries. We performed this comparison for three related molecular databases: ANI-1,^76^ QM9,^11^ and GDB-13.^77^ To ensure the comparison, we first recreated the chemical space corresponding to each database using our method, replicating the main compositional constraints regarding the elements and atom counts. The specific conditions for each database are ANI-1 (H, C, N, and O; ≤8 heavy atoms), QM9 (H, C, N, O, F; ≤9 heavy atoms), and GDB-13 (H, C, N, O, S, and Cl; ≤13 heavy atoms). GDB-13 has additional restrictions on stoichiometries and graph motifs to bias toward specific application domains, which we explicitly did not consider here, as we aim to compare to less biased chemical spaces for novelty discovery.

All molecules from the database were grouped according to their chemical formulas, resulting in frequency distributions of each stoichiometry. Sorting the stoichiometries according to their size in the reference chemical space allows for calculating a well-defined cumulative count of compounds as in Figure 2B. Since databases are meant to form a subset, they are naturally smaller in total count than the underlying space. To account for this, the cumulative counts were normalized by dividing the cumulative counts by the total number of compounds in either space or the database. This transforms the cumulative counts into cumulative distribution functions (CDFs).

Since CDF is a standard way to represent distributions, a wide array of comparison metrics exist. We evaluated the Kolmogorov–Smirnov (KS) test^78^ and the Kullback–Leibler Divergence (KL),^79^ as they have found applications in related works.^80–82^ The KS statistic is a nonparametric test of the equality of continuous, one-dimensional probability distributions. It measures the maximum distance between the CDF values of two samples. Here, those are the chemical space and the database. A lower KS value indicates that the two distributions are closely aligned over the full support, with zero being the ideal value for identical samples. The KL­(*P*∥*Q*) divergence instead shows how one probability distribution diverges from a second distribution.

11 $$KL=\sum_{x\in X}P\left(x\right)\;\log\frac{P\left(x\right)}{Q\left(x\right)}$$

This asymmetric metric captures how much information is lost when *Q* is used to approximate *P*, where *P* represents the chemical space and *Q* is the database distribution. We found KS and KL to agree in their assessment of the similarity of the databases and their underlying chemical space but show results for KS only. This is because the KL metric contains undefined terms if one stoichiometry *x* from the chemical space X is not present in the database, since then *Q*(*x*) = 0 in eq 11, which in practice requires assuming some vanishingly small value for *Q*(*x*), which then inflates the metric and makes it harder to compare between different databases of different chemical spaces. KS does not suffer from this issue, and due to its normalization to the interval [0,1], it is easier to interpret.

Figure 4 shows a) how close existing databases are to their underlying chemical space (see also Table 3 and b) which sampling fraction is a lower bound to representatively capture the distribution of stoichiometry diversity in a database. It is helpful to see not only how well a database represents the underlying target space but also by how much a database could be further reduced while still achieving the same representativeness compared to the full database. Smaller databases would allow for higher-quality reference calculations and, therefore, are generally more desirable. We achieve this by random subsampling of the databases in question.

**Figure 4:**
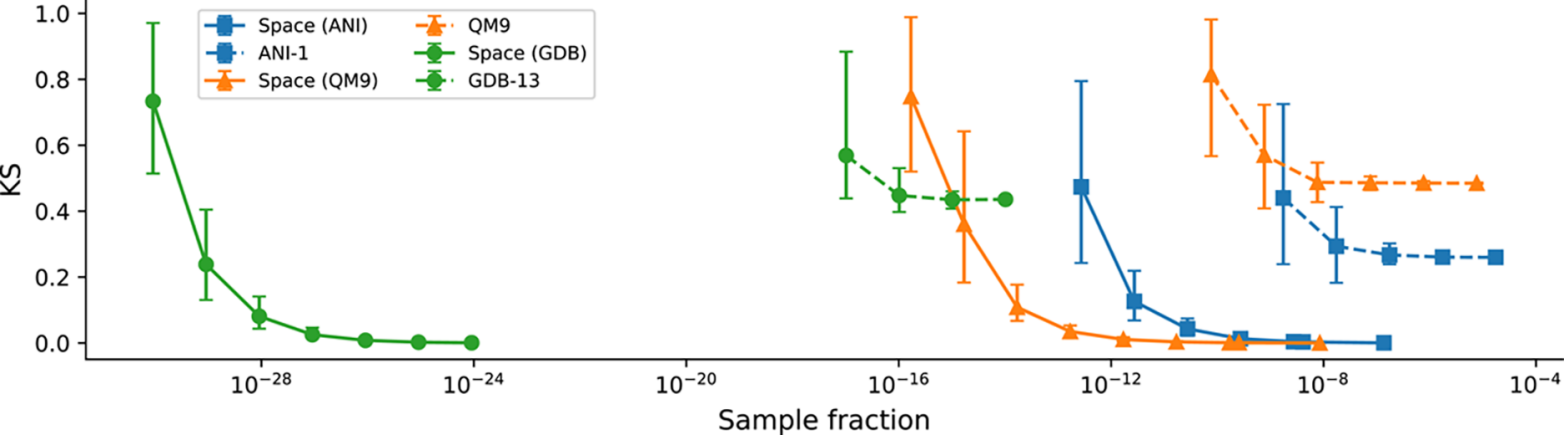
Kolmogorov–Smirnov (KS) statistic as a function of the sampled fraction of the corresponding chemical space. Solid lines represent comparisons between the full space and randomized subspaces thereof. Dotted lines indicate comparisons between subsets sampled from databases (ANI-1, QM9, GDB-13) and the full chemical space for that given database for QM9 and ANI-1. Note that for GDB-13, the lines do not extend across the full range of sampling fractions. Marker shapes distinguish data sets.

**Table 3:** Number of Molecules in the Original Databases (DB Size) and Corresponding Estimated Chemical Space Sizes, along with Kolmogorov–Smirnov (KS) and Kullback–Leibler (KL) Divergence Scores

data set	DB size	chemical space size	KS	KL
ANI-1	5.75 × 10^4^	3.663 × 10^6^	0.260	1.291
QM9	1.31 × 10^5^	5.897 × 10^7^	0.485	5.141
GDB-13	9.77 × 10^8^	1.100 × 10^15^	0.436	16.389

For the QM9 and GDB13 data sets, the KS values are higher than for ANI-1. This behavior is expected given the broader chemical diversity and larger size of these data sets. Higher KS scores reflect the inclusion of valid stoichiometries that are not represented in the original data sets. The small difference between QM9 and GDB13 is a consequence of them being related: QM9 is obtained by further filtering GDB13 to those molecules which converged to a DFT minimum using a particular workflow; therefore, naturally, some of the worst scores of QM9 can be explained by the selection process of which molecular graphs are stable. As discussed above, this only explains part of the QM9 score, as that database is also missing practically relevant molecules. KL scores allow no direct comparison between databases of different chemical spaces as the KL divergence is particularly sensitive to mismatches in the tails of the distributions,^81^ which affects nonsampled low-probability stoichiometries. These outliers increase the referent distance without necessarily compromising the representativeness of the core distribution.

Both the QM9 and the ANI-1 data set implicitly contain three biases: (i) the systematic bias from the selection rules in the GDB enumeration toward graphs which are cheaper to enumerate and likely to follow expectations for stable compounds, since GDB is the foundation for both databases, (ii) the desirable chemical bias toward stable molecules, and (iii) the undesired bias toward molecules that the chosen computational workflows of the databases could deal with. RRS does not suffer from the first and the last bias but lacks any kind of stability information. Therefore, filtering for stability is required when using RRS to build chemical databases: without that, RRS is representative of the possible molecular topologies and not of stable molecular topologies. This, however, requires future research into a highly reliable ab initio workflow, as otherwise, the same biases as in, e.g., QM9 and ANI, would be reintroduced.

Interestingly, the generated chemical spaces required significantly smaller subset sizes to approximate the overall distribution with high fidelity. As a result, the generated spaces can be characterized more efficiently with fewer samples, a useful property for downstream applications such as virtual screening^83^ or training data selection.^22^

Since RRS never builds a full list of all possible molecular graphs, the memory requirements are negligible. The full database of all molecule counts requires 13 MB. Figure 5 shows the compute cost for the case of asking for 100 random molecules for a given stoichiometry using a single core. We compare this to surge, where one would first build a full list of all molecular graphs to then randomly subsample. With only weak dependence on the number of atoms, RRS samples roughly one molecule per second. For small stoichiometries, that is, slower than complete enumeration (e.g., for the C_4_ series in Figure 5), but for larger stoichiometries, the cost to build the full list scales exponentially, while RRS remains roughly constant. For example, for C_12_H_16_, the performance is comparable; for C_14_H_16_, RRS is already more than 100 times faster than enumeration. Note that RRS is trivially parallelizable: any sample is fully independent, so the throughput scales linearly with the number of cores. The largest atom count we have sampled with RRS in Figure 5 is 44 atoms, which takes about 0.8 s per molecule—a vanishingly small cost compared to molecular property calculations.

**Figure 5:**
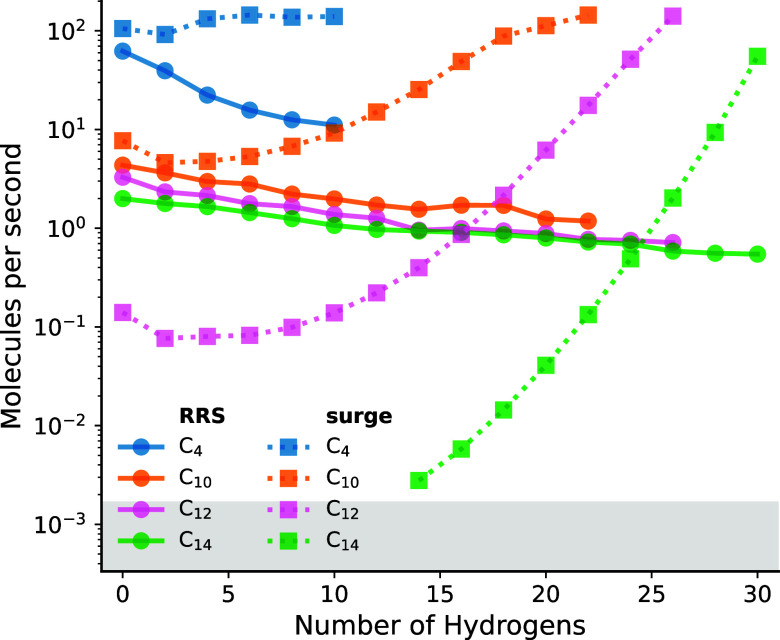
Speed of molecule generation on a single core using either this work (RRS, circles) or the graph enumeration tool surge (squares). For the surge, runs which are too slow (inside the gray area) have been canceled.

## 4 Conclusions

Whenever we make data-driven statements in an attempt to characterize the global behavior of chemical space such as structure–property relationships, then the fundamental question is whether those are valid for single regions in chemical space or whether this has general predictive power. Either of them would be useful, but it is important to be able to distinguish the cases. Our method potentially allows one to put any such data-driven statement on a more rigorous footing by sampling molecules from a given chemical space, making sure to consider all of them without rejection and then testing whether a statement is correct on the sampled molecules. Potentially, this could either uncover a bias in existing databases or generative model output and analysis or prove the lack thereof. In both cases, this enables work toward improved understanding of chemical space as a whole, moving away from considering constituent molecules on a case-by-case basis. Statistical analysis of the generated molecules may help to identify additional trends and structural properties of the chemical space.

One of the main advantages of machine learning is the existence of formal guarantees of how a model must behave or must improve if additional data points are provided. These formal guarantees, however, usually require certain good behavior of the underlying target function such as independent and identically distributed training data. It is not clear whether current databases fulfill that requirement since ensuring a certain distribution of data points is rarely enforced in database generation. Coverage of chemical space becomes more important with multilevel machine learning methods, and with our approach, relevant coverage becomes computationally feasible even for large spaces.

The main limitations of our approach lie in a) the need for a prebuilt database of average path lengths, even if it is shared among all researchers, and b) the approximative nature of the exploited correlations, which renders the resulting samples only approximately uniformly distributed over chemical space. Finally, some of the resulting graphs will not admit a stable minimum energy geometry; future work may address this by introducing additional weights for stoichiometries.

Finally, our work helps to address a more subtle effect regarding machine learning databases: many of them are quite old (more than ten years^11^) but are still used for benchmarking. This means that over time, there is a risk that we do not obtain new models that are of lower generalization error but rather models which, if trained on these databases, can predict the same benchmark databases well. This is a necessary consequence of using the same benchmark databases for many iterations of different models, even if each of these is designed and cross-validated following best practices. This means that in order to test generalization error, we also need test data to be a moving target, as guaranteed to be unseen data.^84^ Using the randomized sampling approach, this can be achieved easily because the space from which to sample is virtually unlimited. In this approach, one could in regular intervals obtain small sets of unseen data that then afford a more truthful approximation to the generalization error in quantum chemistry.
